# Gender minority stress and access to health care services among transgender women and transfeminine people: results from a cross-sectional study in China

**DOI:** 10.1186/s12879-021-06782-5

**Published:** 2021-10-14

**Authors:** Yongjie Sha, Willa Dong, Weiming Tang, Lingling Zheng, Xi Huang, Kathryn E. Muessig, Joseph D. Tucker

**Affiliations:** 1 University of North Carolina Project – China, 7 Lujing Road, Guangzhou, 510091 Guangdong China; 2 Gillings School of Global Public Health, University of North Carolina at Chapel Hill, Chapel Hill, NC USA; 3 School of Medicine, University of North Carolina at Chapel Hill, Chapel Hill, NC USA; 4 Guangzhou Women and Children’s Medical Center, Guangzhou, China; 5 Trans Well-being Team, Guangzhou, China; 6 Faculty of Infectious and Tropical Diseases, London School of Hygiene & Tropical Medicine, London, UK

**Keywords:** Transgender, Gender diverse, Gender minority stress, Sexual health, Gender-affirming care, China

## Abstract

**Background:**

Transgender and gender diverse individuals often face structural barriers to health care because of their gender minority status. The aim of this study was to examine the association between gender minority stress and access to specific health care services among transgender women and transfeminine people in China.

**Methods:**

This multicenter cross-sectional study recruited participants between January 1st and June 30th 2020. Eligible participants were 18 years or older, assigned male at birth, not currently identifying as male, and living in China. Gender minority stress was measured using 45 items adapted from validated subscales. We examined access to health care services and interventions relevant to transgender and gender diverse people, including gender affirming interventions (hormones, surgeries), human immunodeficiency virus (HIV) and sexually transmitted infections (STIs) testing, pre-exposure prophylaxis (PrEP) and post-exposure prophylaxis (PEP). Multivariable regression was used to measure correlations between gender minority stress and access to health care service.

**Results:**

Three hundred and twenty-four people completed a survey and data from 277 (85.5%) people were analyzed. The mean age was 29 years old (standard deviation [SD] = 8). Participants used hormones (118/277, 42.6%), gender affirming surgery (26/277, 9.4%), HIV testing (220/277, 79.4%), STI testing (132/277, 47.7%), PrEP (24/276, 8.7%), and PEP (29/267, 10.9%). Using gender affirming hormones was associated with higher levels of discrimination (adjusted odds ratio [aOR] 1.41, 95% confidence interval [CI] 1.17–1.70) and internalized transphobia (aOR 1.06, 95%CI 1.00–1.12). STI testing was associated with lower levels of internalized transphobia (aOR 0.91, 95%CI 0.84–0.98).

**Conclusions:**

Our data suggest that gender minority stress is closely related to using health services. Stigma reduction interventions and gender-affirming medical support are needed to improve transgender health.

**Supplementary Information:**

The online version contains supplementary material available at 10.1186/s12879-021-06782-5.

## Background

Many transgender and gender diverse individuals have unmet health needs [1]. Transgender individuals have a high prevalence of HIV infection [2]. Despite the high HIV prevalence, transgender and gender diverse individuals have low uptake of HIV prevention services [3, 4]. In addition, unmet needs for gender affirmation may increase sexual health risks for some transgender populations [5]. Gender affirmation refers to the process of receiving recognition and support for one’s gender identity and expression, which is a key determinant of health and well-being for transgender and gender diverse people [1, 6–9]. Gender affirmation can involve medical interventions and/or changing one’s name on legal documents [10, 11]. Many transgender and gender diverse people lack access to medical gender affirmation or essential sexual health services. This disparity is related to stigma against these populations.

Stigma against transgender and gender diverse people refers to institutional, interpersonal, and individual processes that systemically disadvantage them socially, economically, and politically [5, 12, 13]. Transgender and gender diverse people may experience excess stress because of stigma based on identifying as a gender minority [14]. The gender minority stress and resilience (GMSR) model includes stressors related to discrimination, rejection, victimization, negative expectations for the future, internalized transphobia, and gender identity concealment [15]. Minority stress places gender and sexual minorities at risk for a number of negative physical and mental health outcomes [16, 17]. Research has suggested three major pathways that stigma and discrimination impact health: psychosocial stress, access to health and social resources, and violence and bodily harm [18]. Yet, these pathways are not always independent from each other in contributing to health outcomes [19]. For example, research among sexual minority men in the US has suggested that psychological stress has an impact on their engagement in HIV prevention [19]. Understanding the role of discrimination and psychological stress in access to sexual health prevention services is important because prevention is an essential part of comprehensive HIV control. For transgender populations in particular, discrimination and psychological stress can impact decisions to access gender affirmation medical care when other gender affirmation sources are restricted, which has powerful implications for their well-being [6]. However, no studies have assessed these associations in low- and middle-income countries (LMICs), including China.

Previous data suggested that Chinese transgender and gender diverse people experienced high levels of discrimination, victimization, and rejection [20, 21]. Current laws and policies restrict transgender people’s access to gender affirming services, including gender affirming medical care. Transgender and gender diverse people in China also lack protections against employment discrimination and sexual harassment [22]. Difficulties in finding gender affirming medical services push many to seek informal interventions or self-care for gender affirmation (hormones and surgeries) [22]. Additionally, Chinese transgender and gender diverse people may be less likely to test for HIV or sexually transmitted infections (STIs) compared to cisgender HIV key populations such as men who have sex with men (MSM) [23]. Few studies exist on pre-exposure prophylaxis (PrEP) or post-exposure prophylaxis (PEP) uptake in this population [24–26].

The purpose of this cross-sectional study was to examine gender minority stress and access to select health care services among transgender women and transfeminine people in China, focusing on access to gender affirming medical services and interventions (hereafter “gender affirming medical care”) and sexual health services.

## Methods

### Study design and population

This cross-sectional study was conducted in nine cities across eight provinces in China. Study cities were selected because of their relatively large numbers of transgender women and transfeminine people and local community-based organizations (CBOs) providing services to this population. Partnering CBOs shared study announcements on social media and in-person at outreach events and entertainment venues (mainly night clubs). Participants were recruited in-person before February 2020 and then in-person and online during and after February 2020 aligning with local regulations under the COVID-19 pandemic. Inclusion criteria were: 18 years old or older; assigned male at birth; currently identify as a woman, or as a non-binary or gender non-conforming person; and typically residing in one of the study cities.

### Data collection

Participants completed the survey between January 1st and June 30th 2020. Eligible participants completed a self-administered computer-assisted survey. The survey collected data on sociodemographic information, gender identity, gender affirming medical care use (hormones, surgeries), HIV testing history, STI testing history (gonorrhea, chlamydia, syphilis, human papilloma virus [HPV] or condyloma acuminate, Herpes simplex virus 2 [HSV-2]), HIV serostatus, PrEP use, PEP use, gender minority stress and resilience, and sex work. We modified the two-step method to collect gender identity data [1]. Participants first selected their assigned sex at birth, and then gender identity from response options based on previous formative research and community expert feedback (“Transgender women”, “CD”, “Yao or TS”, “Women”, “Gender non-conforming, nonbinary”, and “other, please describe”) (unpublished). CD, TS, and Yao are local, reclaimed and contested identity terms [27]. Except for screening questions, participants were allowed to skip questions or choose “refuse to answer”. Participants automatically received a unique eight-digit survey identification number after completing the online questionnaire (Sojump, Shanghai, China). All participants were required to contact CBO staff and verify questionnaire submission using their survey identification number to receive payment. Eligible participants who completed the survey received $15.

Participants who visited CBOs that routinely offered HIV testing services were also asked whether they would like to receive HIV testing. CBO staff members obtained informed consent and performed the test following their standard protocol. Results were reported in an additional Sojump questionnaire, as well as the location and time of the test, survey identification number, and consent.

We restricted eligible responses as those that took at least 5 min as the survey hosted over 200 questions. Survey completion time was used to supplement other measures to prevent careless responses, such as attention checks [28]. CBOs also verified each questionnaire submission using survey identification numbers and contacted those suspected of submitting fraudulent responses.

### Measures

#### Sociodemographic

Participants reported sociodemographic characteristics including age, ethnicity, housing, marital status, education, monthly income (CNY/USD = 6.5), sexual attraction, gender identity, gender identity disclosure, whether they currently live as the gender they identify as, and sex work involvement.

#### Gender minority stress

We adapted the subscales from the GMSR measure to assess gender minority stress [15]. These subscales measured participants’ adverse experiences related to gender identity and perceptions about their own identity and transgender community. The measure includes seven factors: discrimination (5 items with total values ranging from 0 to 5, see Additional file 1: Online Appendix for a full list of items), rejection (6 items, 0–6), victimization (6 items, 0–6), non-affirmation of gender identity (6 items, 0–18), internalized transphobia (8 items, 0–24), negative expectation for the future (9 items, 0–27), and nondisclosure of gender identity (5 items, 0–15). Items in the discrimination, rejection, and victimization subscales are scored 0 for “No” and 1 for “Yes”. The non-affirmation of gender identity, internalized transphobia, negative expectation for the future, and nondisclosure of gender identity subscale items are scored on a 4-point Likert scale: 0 (strongly disagree), 1 (somewhat disagree), 2 (somewhat agree), and 3 (strongly agree). All subscales were modified to omit recency and whether or not the stressors were experienced before age 18 to reduce participant burden.

The subscales were adapted to the Chinese context following recommendations for cross-cultural adaptation of self-reported measures [29]. The subscales were translated into Chinese by two graduate students with expertise in public health and social science who were bilingual in English and Chinese, back translated into English by another bilingual public health graduate student, and reviewed by two academic and two community experts in transgender health in China. Ten cognitive interviews with transgender women and transfeminine people in two cities were conducted in Mandarin by a member of the study team (WD) and research assistants prior to pretesting. Feedback from the cognitive interviews were synthesized for each item. Scale items, instructions, and response options were modified based on these activities as well as prior formative and qualitative research (unpublished). Finally, pretesting with transgender women and transfeminine people was conducted prior to survey implementation.

#### Access to health care services

We included gender affirming medical care use (hormones, surgeries), HIV testing, STI testing, PrEP use, and PEP use to examine participants’ access to health care services. In China, few hospitals offer gender affirming medical care, while HIV and STI testing can be accessed readily at hospitals, CBO-led testing clinics, or through self-testing kits purchased online. PrEP and PEP are only available at select infectious disease hospitals and require a prescription. Since PrEP was only approved in China in August 2020, participants in our survey likely accessed PrEP from informal sources or clinical trials. Similarly, transgender and gender diverse people in China may access gender affirming medical care informally due to low availability and high price. We did not distinguish between formal and informal access to these health care resources.

Lifetime HIV testing and PEP use were examined using single items with yes/no response options. STI testing in the past year was assessed where testing for gonorrhea, chlamydia, syphilis, HPV, or HSV-2 was counted as ever tested. For lifetime hormone intervention history, gender affirming surgery history, and PrEP use history, willingness was assessed in addition to yes/no response options. In this analysis, all responses other than ever use for gender affirming interventions and PrEP use were considered as never used.

### Statistical analysis

Frequencies of categorical variables regarding sociodemographic characteristics and access to health care services were calculated as the proportion of participants. Mean scores of each gender minority subscales were calculated. Higher scores reflected a higher degree of minority stress. Cases with complete responses for the subscale of interest were included. Missing values were excluded from analysis. Multivariable regression (stepwise) was performed to assess associations between each gender minority stress subscale and each type of health care service. We adjusted for income, education, sexual attraction, and gender identity for analyses related to sexual health services access [30–33]. Additionally, we adjusted for whether the individual currently lives as their identified gender for models related to gender affirming medical care use. Adjusted odds ratios (aOR) were reported and the Wald 2-sided 95% confidence interval (CI) was used. A 2-sided P value < 0.05 was considered a statistically significant difference for all comparisons. All analyses were performed using R 3.6.3 (R Core Team).

This study was approved by institutional review boards at the University of North Carolina at Chapel Hill and Southern Medical University-Dermatology Hospital. All participants provided informed consent by checking a box on a self-administered online informed consent form indicating their agreement to participate in the study.

## Results

Overall, 324 participants completed the survey. After excluding 28 submissions that were under 5 min, 18 unverified submissions from the same IP address, and one response from an individual under 18 years old, we included 277 participants for analysis.

### Sociodemographic characteristics of participants

The mean age of the participants was 29 years old (Table 1). Most participants were never married (89%), attracted to men (80.9%), and self-identified as transgender women or women (58.8%). For those who identified otherwise, 37 (13.4%), 37 (13.4%), and 39 (14.1%) self-identified as “gender non-conforming, nonbinary”, “CD”, and “Yao or TS”, respectively. The majority had disclosed their gender identity to at least one other person (207/275, 75.3%), among whom most had disclosed to health workers (155/207, 74.9%). One hundred sixty-six participants (60.1%) currently live as their self-identified gender for most of the time. Finally, one hundred sixteen (42.2%) participants had ever exchanged sex for money in their lifetime.

**Table 1:** Sociodemographic characteristics of transgender women and transfeminine people in China, December 2019–June 2020 (N = 277)

Characteristics	N (%)
Age (n = 253)*	29.04 (7.74)
Housing	
Live alone owned or rent	163 (58.8)
Live with someone else	62 (22.4)
Live at workplace or school	38 (13.7)
Other	14 (5.1)
Marital status (n = 276)*	
Never married	243 (89.0)
Engaged or married	10 (3.7)
Separated or divorced	23 (8.4)
Widowed	0 (0.0)
Education	
High school or below	126 (45.5)
Some college	68 (24.5)
College/Bachelors or above	83 (30.0)
Annual income	
No greater than USD$5538	75 (27.1)
Greater than $5538 and no greater than $11,077	129 (46.6)
Greater than USD$11,077	73 (26.3)
Sexual attraction	
To women	15 (5.4)
To men and women	23 (8.3)
To men	224 (80.9)
To no one	3 (1.1)
Unsure or other	12 (4.3)
Gender identity	
Transgender woman	104 (37.5)
CD	37 (13.4)
Yao or TS	39 (14.1)
Women	59 (21.3)
Gender non-conforming, nonbinary	37 (13.4)
Other	1 (0.3)
Currently live as self-identified gender for most of the time (n = 276)*	
Yes	166 (60.1)
No	110 (39.9)
Disclosed identity (n = 275)*	
Yes	207 (75.3)
No	68 (24.7)
Disclosed identity to health workers	
Yes	155 (74.9)
No	52 (25.1)
Ethnicity	
Han	217 (80.3)
Other	53 (19.6)
Lifetime sex work involvement	
Yes	116 (42.2)
No	159 (57.8)

CD, Yao, and TS are local transfeminine identity terms

*Numbers may not add up to 277 due to missing values

### Access to health care services

Table 2 presents data on participants’ access to health care services. For gender affirming interventions, 42.6% received hormone interventions while 9.4% underwent any gender affirming surgical procedures. Most participants (79.4%) had ever received HIV testing while less than half (47.7%) had ever tested for STIs. For PrEP and PEP, 8.7% and 10.9% had ever received these interventions, respectively. The HIV prevalence among those ever-tested was 9.1% (20/220).

**Table 2:** Access to health care services among transgender women and transfeminine people in China, December 2019–June 2020 (N = 277)

Health care services	N (%)
Hormone intervention history	
Have or currently undergoing	118 (42.6)
Never	159 (57.4)
Gender affirming surgery	
Have or currently undergoing	26 (9.4)
Never	251 (90.6)
HIV testing	
No	57 (20.6)
Yes	
Lifetime HIV testing	220 (79.4)
Lifetime facility-based HIV testing	190 (86.4)
Lifetime HIV self-testing	150 (68.5)
STIs testing in the past year	
No	145 (52.3)
Yes	
Gonorrhea	23 (8.3)
Chlamydia	15 (5.4)
Syphilis	124 (44.8)
HPV	33 (11.9)
HSV2	8 (2.9)
PrEP use	
Yes	24 (8.7)
No	252 (91.3)
PEP use	
Yes	29 (10.9)
No	238 (89.1)
Living with HIV	20 (9.1)

### Experiences with gender minority stress

Table 3 shows the mean scores of gender minority stress subscales. Additional file 1 reports scores for each item. Participants experienced high levels of non-affirmation of gender identity (mean, M = 11.92) and scored the highest on item 2 (“I have difficulty being perceived as my gender.”) (M = 2.10). Many participants (80%) also reported being verbally harassed or teased (victimization subscale, item 1).

**Table 3:** Gender minority stress subscale scores among transgender women and transfeminine people, January–June 2020 (N = 277)

Subscales (number of items)	Range	Completion N (%)	Alpha*	Total score Mean ± SD^¶^
Discrimination (5)	0–5	273 (98.6)	0.72	2.57 ± 1.65
Rejection (6)	0–6	271 (97.8)	0.80	2.68 ± 2.08
Victimization (6)	0–6	246 (88.8)	0.84	2.64 ± 2.08
Non-affirmation of gender identity (6)	0–18	276 (99.6)	0.88	11.92 ± 3.68
Internalized transphobia (8)	0–24	274 (98.9)	0.93	12.93 ± 5.40
Negative expectations for future (9)	0–27	276 (99.6)	0.93	15.37 ± 5.70
Non-disclosure of gender identity (5)	0–15	274 (98.9)	0.89	9.15 ± 3.41

*Cronbach’s alpha of internal consistency based on standardized items

### Gender minority stress and access to health care services

Table 4 presents the associations between gender minority stress and participants’ access to health care services based on five different models with one model for each outcome. All variables included in the regression models are presented. Ever using hormones was associated with higher levels of discrimination (aOR 1.41, 95%CI 1.17–1.70) and internalized transphobia (aOR 1.06, 95%CI 1.00–1.12). Having undergone gender affirming surgery was associated with high levels of discrimination, with an adjusted odds ratio of 1.44 (95% CI 1.06–1.97).

**Table 4:** Associations between gender identity stress and access to health care services among transgender women and transfeminine people in China, December 2019–June 2020

Variables	aOR	95% CI
Hormone intervention history (ref = never)^a^		
Have or currently undergoing		
Discrimination	1.41	1.17–1.70
Internalized transphobia	1.06	1.00–1.12
Gender affirming surgery (ref = never)^a^		
Have or currently undergoing		
Discrimination	1.44	1.06–1.97
Non-disclosure of gender identity	0.87	0.77–0.99
HIV testing (ref = no)^b^		
Discrimination	0.70	0.54–0.92
STIs testing in the past year (ref = no)^b^		
Internalized transphobia	0.91	0.84–0.98
Negative expectation for future	1.12	1.04–1.19
PEP use (ref = no)^b^		
Non-disclosure of gender identity	1.19	1.05–1.35

^a^Models were adjusted with education, income, sexual attraction, gender identity, and whether the individual currently live in self-identified gender for most of the time

^b^Models were adjusted with education, income, sexual attraction, and gender identity

HIV testing was associated with lower levels of discrimination (aOR 0.70, 95%CI 0.54–0.92). STI testing was associated with lower levels of internalized transphobia (aOR 0.91, 95%CI 0.84–0.98) but higher levels of negative expectations for future (aOR 1.12, 95%CI 1.04–1.19). PEP use was associated with higher levels of stress related to gender identity non-disclosure (aOR 1.19, 95%CI 1.05–1.35).

## Discussion

**Fig. 1:**
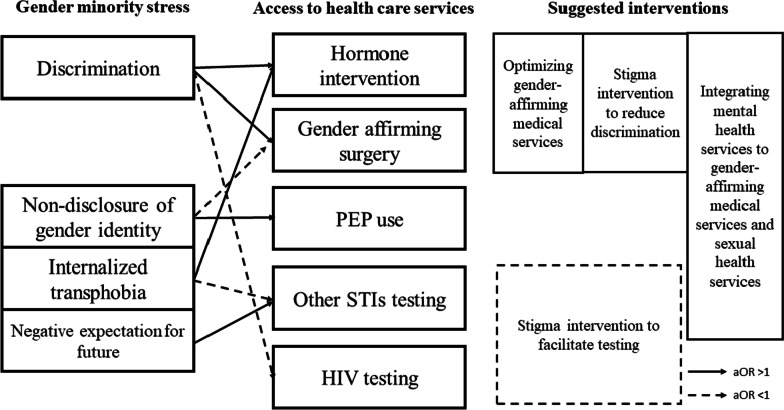
Gender minority stress, access to health care services, and suggested interventions based on a sample of 277 transgender women and transfeminine people in China, 2020

This study examined gender minority stress and access to health care resources among transgender women and transfeminine people in China. We identified low uptake of gender affirming medical care (hormones, surgeries), STI testing, PrEP and PEP. Discrimination and internalized transphobia are likely barriers to HIV and STI testing, though some gender minority stressors are also associated with higher uptake of some health care services (Fig. 1). More research on gender minority stress and discrimination among transgender and gender diverse people is needed. This study contributes to our understanding of the role of minority stress in engaging health care services.

We found low STI testing rates among study participants, which is consistent with prior studies among transgender women globally, including in China [23, 34]. Our findings suggest that internalized transphobia is a barrier to STI—but not HIV—testing among Chinese transgender women and transfeminine people. These results could be due to the fact that STI testing usually requires going to a hospital where providers may not be knowledgeable about transgender health while HIV testing is accessible at CBOs that often serve transgender and gender diverse people. There are ongoing efforts in China to expand STI testing among other key populations in China, but fewer programs focused on transgender and gender diverse people [35, 36]. Interventions that address or take into consideration internalized transphobia such as self-testing or offering testing at venues reaching transgender and gender diverse people (CBOs, entertainment venues), training healthcare providers, and establishing transgender-friendly clinics in the vein of MSM-friendly clinics may increase STI testing accessibility to Chinese transgender and gender diverse populations.

We found that transgender people who experienced discrimination and internalized transphobia were more likely to use gender affirming hormones. Previous data from the US suggested that these stressors can hinder accessing gender affirmation medical care [37]. This finding is aligned with the Gender Affirmation Framework suggesting discrimination and internalized transphobia may cause psychological distress that increase need for gender affirmation [6]. For example, those who experienced low visual conformity to dominant gender presentation norms are subject to discrimination and violence and might be more willing to receive hormone intervention [13]. However, this was not a longitudinal study and this relationship between discrimination and using gender affirming hormones could also be explained related to participants encountering discrimination in the process of getting care, informal access to hormones, or internalization of beauty standards. Future research is needed to assess the relationship between gender minority stress and gender affirmation services in resource-limited settings. Research should also explore the ways Chinese transgender women and transfeminine people seek gender affirmation given the low availability of gender affirming medical care. More gender affirming medical care and other gender affirmation services (e.g., easier legal process for changing gender markers on documents) in China are needed. Multi-level interventions, including nationwide comprehensive gender identity education, gender-affirming health care capacity building, community empowerment and advocacy and psychological counseling, may also be helpful to reduce stress related to discrimination and internalized transphobia.

Similarly, the positive association between gender minority stressors and STI testing uptake may reflect that gender minority stress is closely associated with HIV prevention among study participants. Previous research on sexual minority men in the US suggested that psychological distress was associated with higher levels of HIV prevention engagement when felt stigma was low [19]. Although our study did not directly measure felt stigma, our findings support the hypothesis that minority stress could mediate the relationship between stigma and engagement in sexual health services [38].

## Research and policy implications

Our findings have research and policy implications. First, our study suggests the importance of gender minority stressors to health outcomes given the associations we found with healthcare service access. More research is needed to examine the processes that connect stigma, gender minority stress, and health outcomes among transgender and gender diverse people in China. Second, stigma reduction measures targeting discrimination and internalized transphobia would be helpful to decrease barriers to HIV and STI testing. Few stigma reduction interventions are documented in the peer-reviewed literature due to the scarcity of research on transgender and gender diverse people and the political sensitivity of this group in China. Prior efforts from community-based organizations in China include transgender-affirmative mental health practice training for clinical professionals. Future interventions could include online support groups to reduce internalized transphobia and foster resilience; psychological counseling interventions to address internalized transphobia; training in cultural competence for primary care and sexual health physicians [13, 39, 40]. Third, interventions should address the high levels of minority stress related to accessing health services and interventions. Increasing the quality of gender affirming medical care, establishing standard operating protocols for medical professionals, and removing barriers to these services are important next steps. In terms of stressors associated with sexual health care access, we suggest integrating mental health services with sexual health interventions and care [41–43]. Sexual health services could integrate mental health components by directly providing counseling services, providing referrals to psychological services, and organizing community engagement activities for the transgender and gender diverse people [42, 44, 45].

## Limitations

Our study should be read with limitations. First, this cross-sectional study is unable to establish temporality or explain associations. Additional research (longitudinal and qualitative) is needed on transgender and gender diverse people’s experiences of health care service access in order to reduce minority stress. Second, most of the partnering community-based organizations were groups providing HIV testing services, as there were no transgender-specific CBOs that we knew of in most study cities. Collaborating organizations promoted the study through social media platforms and in-person outreach. This recruitment process could introduce selection bias. Compared to samples collected in previous studies [20, 25], our participants have similar sociodemographic characteristics with regards to age, education, and income. However, due to the overall marginalization of transgender and gender diverse people in China, people who were older, less familiar with online survey, or less engaged in community-based HIV services may have been undersampled. A prior study in China [27] has demonstrated that it is feasible to use methods such as respondent-driven sampling to recruit participants. Building long-term, respectful partnerships with community-based organizations is key to reaching diverse participants across China. Third, our survey did not distinguish between informal and formal access to healthcare services such as hormone interventions. It is likely that differing gender minority stressors are relevant depending on access channels.

## Conclusions

Gender minority stress is correlated with transgender gender diverse people’s access to health care services in different ways. Discrimination and internalized transphobia are barriers to HIV and STI testing, while some gender minority stressors are associated with higher engagement in gender affirming medical care and sexual health prevention services. Future research in China should investigate the pathways that connect stigma, minority stress, and health outcomes to better understand transgender health inequalities and to inform interventions. Future policy is needed to prioritize providing gender-affirming medical services for Chinese transgender individuals and include mental health services in transgender health care delivery.

## Supplementary Information

**Additional file 1. **Scores of gender minority stress subscales by item, N = 277, December 2019–June 2020.

## Data Availability

The data sets used and/or analyzed during the current study may be made available upon reasonable written request to the corresponding author.

## Abbreviations

HIV: Human immunodeficiency virus
GMSR: Gender minority stress and resilience
US: The United States
LMIC: Low and middle-income country
STI: Sexually transmitted infection
MSM: Men who have sex with men
PrEP: Pre-exposure prophylaxis
PEP: Post-exposure prophylaxis
CBO: Community-based organization
HPV: Human papilloma virus
HSV-2: Herpes simplex virus 2
CD: Cross-dresser
TS: Transsexual
aOR: Adjusted odds ratio
CI: Confidence interval
