# Supplementary material for: Gender minority stress and access to health care services among transgender women and transfeminine people: results from a cross-sectional study in China
Source: BMC Infect Dis. 2021 Oct 14;21:1065. doi: 10.1186/s12879-021-06782-5 (PMC8514805; doi:10.1186/s12879-021-06782-5)
Supplement: Supplementary file 1 — Additional file 1. Scores of gender minority stress subscales by item, N = 277, December 2019–June 2020. [file 12879_2021_6782_MOESM1_ESM.docx]

**Additional Information**

**Additional file 1**

**Scores of gender minority stress subscales by item, N = 277, December 2019 – June 2020.**

| **Scales** | **Completion N (%)** | **Mean score** |
| --- | --- | --- |
| **Discrimination** | 273 (98.6) | 2.57 |
| 1. I have had difficulty getting medical or mental health treatment (transition-related or other) because of my gender identity or expression. |  | 0.52 |
| 2. Because of my gender identity or expression, I have had difficulty finding a bathroom to use when I am out in public. |  | 0.60 |
| 3. I have experienced difficulty getting identity documents that match my gender identity. |  | 0.67 |
| 4. I have had difficulty finding housing or staying in housing because of my gender identity or expression. |  | 0.27 |
| 5. I have had difficulty finding employment or keeping employment, or have been denied promotion because of my gender identity or expression. |  | 0.51 |
| **Rejection** | 271 (97.8) | 2.68 |
| 1. I have had difficulty finding a partner or have had a relationship end because of my gender identity or expression. |  | 0.62 |
| 2. I have been rejected or made to feel unwelcome by a religious community because of my gender identity or expression. |  | 0.38 |
| 3. I have been rejected by or made to feel unwelcome in my ethnic/racial community because of my gender identity or expression. |  | 0.36 |
| 4. I have been rejected or distanced from friends because of my gender identity or expression. |  | 0.42 |
| 5. I have been rejected at school or work because of my gender identity or expression. |  | 0.47 |
| 6. I have been rejected or distanced from family because of my gender identity or expression. |  | 0.43 |
| **Victimization** | 246 (88.8) | 2.64 |
| 1. I have been verbally harassed or teased because of my gender identity or expression. |  | 0.80 |
| 2. I have been threatened with being outed or blackmailed because of my gender identity or expression. |  | 0.36 |
| 3. I have had my personal property damaged because of my gender identity or expression. |  | 0.41 |
| 4. I have been threatened with physical harm because of my gender identity or expression. |  | 0.40 |
| 5. I have been pushed, shoved, hit, or had something thrown at me because of my gender identity or expression. |  | 0.42 |
| 6. I have had sexual contact with someone against my will because of my gender identity or expression. |  | 0.24 |
| **Non-affirmation of gender identity** | 276 (99.6) | 11.92 |
| 1. I have to repeatedly explain my gender identity to people or correct the pronouns people use. |  | 1.89 |
| 2. I have difficulty being perceived as my gender. |  | 2.10 |
| 3. I have to work hard for people to see my gender accurately. |  | 2.09 |
| 4. I have to be “hypermasculine” or “hyperfeminine” in order for people to accept my gender. |  | 1.88 |
| 5. People don’t respect my gender identity because of my appearance or body. |  | 1.87 |
| 6. People don’t understand me because they don’t see my gender as I do. |  | 2.09 |
| **Internalized transphobia** | 274 (98.9) | 12.93 |
| 1. I resent my gender identity or expression. |  | 1.56 |
| 2. My gender identity or expression makes me feel like a freak. |  | 1.36 |
| 3. When I think of my gender identity or expression, I feel depressed. |  | 1.50 |
| 4. When I think about my gender identity or expression, I feel unhappy. |  | 1.54 |
| 5. Because my gender identity or expression, I feel like an outcast. |  | 1.72 |
| 6. I often ask myself: Why can’t my gender identity or expression just be normal? |  | 1.71 |
| 7. I feel that my gender identity or expression is embarrassing. |  | 1.66 |
| 8. I envy people who do not have a gender identity or expression like mine. |  | 1.89 |
| **Negative expectation for future** | 276 (99.6) | 15.37 |
| 1. If I express my gender identity/history, others wouldn’t accept me. |  | 1.75 |
| 2. If I express my gender identity/history, employers would not hire me. |  | 1.83 |
| 3. If I express my gender identity/history, people would think I am mentally ill or “crazy.” |  | 1.80 |
| 4. If I express my gender identity/history, people would think I am disgusting or sinful. |  | 1.90 |
| 5. If I express my gender identity/history, most people would think less of me. |  | 1.85 |
| 6. If I express my gender identity/history, most people would look down on me. |  | 1.82 |
| 7. If I express my gender identity/history, I could be a victim of crime or violence. |  | 1.60 |
| 8. If I express my gender identity/history, I could be arrested or harassed by police. |  | 1.32 |
| 9. If I express my gender identity/history, I could be denied good medical care. |  | 1.50 |
| **Non-disclosure of gender identity** | 274 (98.9) | 9.15 |
| 1. Because I don’t want others to know my gender identity/history, I don’t talk about certain experiences from my past or change parts of what I will tell people. |  | 1.82 |
| 2. Because I don’t want others to know my gender identity/history, I modify my way of speaking. |  | 1.84 |
| 3. Because I don’t want others to know my gender identity/history, I pay special attention to the way I dress or groom myself. |  | 1.77 |
| 4. Because I don’t want others to know my gender identity/history, I avoid exposing my body, such as wearing a bathing suit or nudity in locker rooms. |  | 1.96 |
| 5. Because I don’t want others to know my gender identity/history, I change the way I walk, gesture, sit, or stand. |  | 1.76 |
